# Phytochemical Characterization of *Veronica officinalis* L., *V. teucrium* L. and *V. orchidea* Crantz from Romania and Their Antioxidant and Antimicrobial Properties

**DOI:** 10.3390/ijms160921109

**Published:** 2015-09-03

**Authors:** Andrei Mocan, Dan Cristian Vodnar, Laurian Vlase, Ovidiu Crișan, Ana-Maria Gheldiu, Gianina Crișan

**Affiliations:** 1 Department of Pharmaceutical Botany, Iuliu Hațieganu University of Medicine and Pharmacy, 23 Ghe. Marinescu Street, Cluj-Napoca 400010, Romania; E-Mails: mocan.andrei@umfcluj.ro (A.M.); gcrisan@umfcluj.ro (G.C.); 2 Department of Food Science, University of Agricultural Sciences and Veterinary Medicine, 3-5 Manăştur Street, Cluj-Napoca 400372, Romania; E-Mail: dan.vodnar@usamvcluj.ro; 3 Department of Pharmaceutical Technology and Biopharmaceutics, Iuliu Hațieganu University of Medicine and Pharmacy, 12 I. Creangă Street, Cluj-Napoca 400010, Romania; E-Mail: gheldiu.ana@umfcluj.ro; 4 Department of Organic Chemistry, Iuliu Hațieganu University of Medicine and Pharmacy, 12 I. Creangă Street, Cluj- Napoca 400010, Romania

**Keywords:** *Veronica officinalis* L., *V. teucrium* L., *V. orchidea* Crantz, antioxidants, phytosterols, antimicrobials

## Abstract

Aerial parts of *Veronica* species are used in Romanian traditional medicine for the treatment of various conditions like kidney diseases, cough, and catarrh, and are known for their wound-healing properties. In the present study, the phenolic and sterolic content and the antioxidant and antimicrobial activities of three *Veronica* species (*Plantaginaceae*), *V. officinalis* L., *V. teucrium* L. and *V. orchidea* Crantz, were studied. The identification and quantification of several phenolic compounds and phytosterols were performed using LC/MS techniques and the main components were *p*-coumaric acid, ferulic acid, luteoline, hispidulin and β-sitosterol. More than that, hispidulin, eupatorin and eupatilin were detected for the first time in the *Veronica* genus. Nevertheless, representatives of the *Veronica* genus were never investigated in terms of their phytosterol content. The antioxidant potential investigated by Trolox equivelents antioxidant capacity (TEAC) and EPR spectroscopy revealed that *V. officinalis* and *V. orchidea* extracts presented similar antioxidant capacities, whilst the values registered for *V. teucrium* extract are lower. Regarding the antimicrobial activity of the investigated species, *Staphylococcus aureus*, *Listeria monocytogenes* and *Listeria ivanovii* were the most sensitive strains with MIC values between 3.9 and 15.62 mg/mL. The results obtained by this study may serve to promote better use of representatives from the genus *Veronica* as antioxidant and antimicrobial agents.

## 1. Introduction

Natural products have been and continue to be a source of inspiration for a substantial portion of human therapeutics. In medicinal plants, secondary metabolites are considered bioactive natural compounds and may ultimately be developed as drugs. In food, they would be defined as phytonutrients without having therapeutic claims but with significant health benefits that can be useful in disease prevention [1]. Interest in developing natural nutritional antioxidants is increasing due to their well documented impact on human health [2], but also due to the fact that synthetic antioxidants have been incriminated as endocrine disrupters or even carcinogenic agents [3]. Medicinal plants rich in polyphenols can retard the oxidative degradation of lipids and improve the quality and nutritional value of food [2]. Considered to be the most frequent antioxidant compounds in human diets, polyphenols possess multiple biological properties [4], making it vital to learn about their amounts and varieties in medicinal plants and natural foods [5,6].

Sterolic compounds occur in a wide range of plant species; both yellow and green vegetables contain appreciable quantities. Phytosterols have demonstrated the ability to block the uptake of cholesterol (to which they are structurally related) and also facilitate its excretion from the body [7]. The most common phytosterols in natural products are β-sitosterol, stigmasterol, and campesterol. They can reduce the atherosclerotic risk and offer protection against cardiovascular diseases, and decrease the risks of breast, prostate and colon cancer [8,9]. Furthermore, phytosterols have anti-inflammatory and immunomodulatory properties [10]. However, all phytosterol intake comes exclusively from the diet, as they cannot be synthesized by humans. It is known that more than 95% of total phytosterol dietary intake is represented by β-sitosterol, stigmasterol and campesterol [8].

The genus *Veronica* L. is the largest genus of the *Plantaginaceae* family, with about 500 species widespread over most of the Northern Hemisphere and in many parts of the Southern Hemisphere; it presents high ecologically diversity, with species growing in aquatic to dry steppe habitats from sea level to high alpine regions. This diversity and the fact that many species have beautiful blue flowers may explain the interest *Veronica* has attracted for a long time [11]. Aerial parts of *Veronica* species are used as traditional medicine for treatment of various inflammatory conditions, including rheumatism [12]. Some of the *Veronica* species, like *Veronica officinalis* L. have a long history of medicinal use as diuretic and diaphoretic agents. In Romanian folk medicine, it has been used for kidney diseases, cough, and catarrh, and was known for its wound-healing properties and its indication in lung diseases and hypercholesterolemia [13,14,15]. The chemical composition of *Veronica* has been previously investigated regarding its iridoid profile [16,17], but no other data was found regarding the phenolic or sterolic profile of Romanian harvested *V. officinalis*, *V. teucrium* and *V. orchidea*. However, recently, a few studies have confirmed that certain *Veronica* species showed noticeable bioactivity such as antibacterial [18], antioxidant [19,20], anti-inflammatory [21] and citotoxic [21,22] activity.

**Figure 1:**
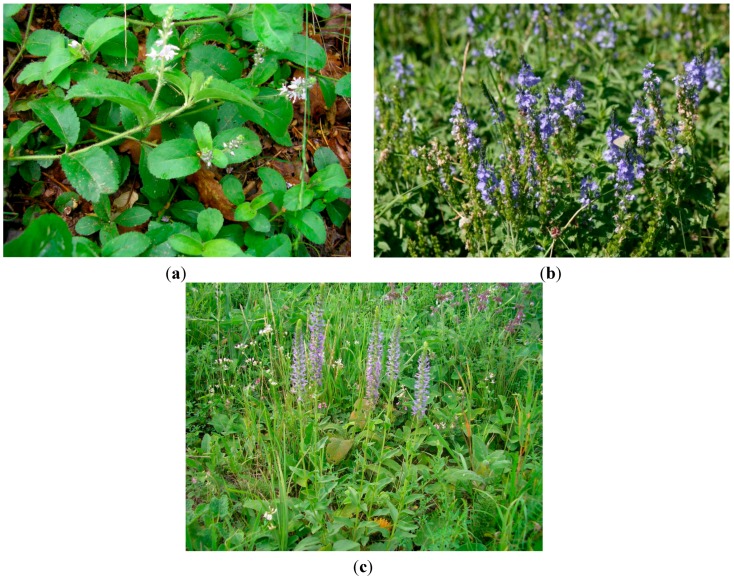
(**a**) *V. officinalis* L.; (**b**) *V. teucrium* L.; (**c**) *V. orchidea* Crantz.

Within this frame, this study aimed to investigate the phenolic and sterolic composition of aerial parts of relevant *Veronica* species (*V. officinalis*, *V. teucrium* and *V. orchidea*) harvested from the Romanian spontaneous flora (Figure 1a–c). The quantitative content of the phenolic or sterolic compounds was estimated using HPLC-MS (high performance liquid chromatography coupled with mass spectrometry) techniques. This study was also intended to explore the antioxidant capacities of the three species by means of spectrophotometry and EPR (electron paramagnetic resonance) spectroscopy. Additionally, the evaluation of the antimicrobial effect was carried out using a microdilution technique.

## 2. Results and Discussion

### 2.1. HPLC-MS Analysis of Phenolic Compounds

In this study the presence and content of different phenolic compounds in 70% ethanolic extracts was studied using an HPLC-MS method which allows the simultaneous detection of several phenolic compounds with a single column pass [23,24,25]. In order to obtain more accurate data on flavonoid glycoside and aglycone concentrations, and to estimate the nature of the hydrolyzed compounds, each sample was analyzed before and after acid hydrolysis [26]. According to the obtained results, the phenolic content varies greatly among tested *Veronica* species, as seen in Table 1.

**Table 1:** The polyphenolic compounds content in the studied samples (μg/g d.w. plant material).

Polyphenolic Compound	*V. officinalis*	*V. officinalis* ^H^	*V. teucrium*	*V. teucrium* ^H^	*V. orchidea*	*V. orchidea* ^H^
Gentisic acid	<0.02	–	<0.02	–	<0.02	–
Caffeic acid	<0.02	–	NF	–	<0.02	–
Chlorogenic acid	<0.02	–	< 0.02	–	<0.02	–
*p*-Coumaric acid	18.25 ± 0.21	85.06 ± 0.09	24.27 ± 0.23	36.91 ± 0.43	14.04 ± 0.05	46.54 ± 1.25
Ferulic acid	31.35 ± 0.25	431.78 ± 8.70	13.66 ± 0.43	54.11 ± 0.08	16.69 ± 0.08	141.57 ± 5.76
Sinapic acid	–	5.52 ± 0.05	–	23.31 ± 1.31	–	8.21 ± 0.03
Quercitrin	87.78 ± 0.51	7.39 ± 0.13	–	–	<0.02	–
Quercetin	–	3.39 ± 0.05	–	–	–	19.35 ± 0.25
Luteolin	36.48 ± 0.18	89.72 ± 3.08	–	6.06 ± 0.05	–	42.01 ± 2.01
Apigenin	6.77 ± 0.03	7.75 ± 0.05	–	7.75 ± 0.03	8.73 ± 0.01	4.81 ± 0.01

Note: NF—not found, below limit of detection. ^H^—hydrolyzed sample. Values are the mean ± SD (*n* = 3). d.w.—dry weight.

Regarding the phenolic acids, *p-*coumaric acid was the most abundant in *V. teucrium* extract (24.27 ± 0.23 μg/g d.w. plant material) and ferulic acid (31.35 ± 0.25 μg/g d.w. plant material) in the *V. officinalis* extract. However, gentisic and chlorogenic acids were identified in all extracts but their amounts were too low to be quantified. The presence of phenolic acids in several *Veronica* species was previously reported by several authors. Thus, Beara *et al*. [12] reported lower amounts of ferulic and *p-*coumaric acids in Serbian harvested *V. teucrium*. The same authors reported the presence of these compounds also in aerial parts of *V. jaquinii* and *V. urticifolia* [12]. Higher amounts of caffeic acid were reported by Barreira *et al.* [27] in *V. montana* and *V. spuria* and by Živković *et al.* [28] in *V. urticifolia*. The increased quantities of phenolic acids after acid hydrolysis suggest the presence of several glycosylated structures in these compounds.

Quercitrin was identified only in *V. officinalis* (87.78 ± 0.51 μg/g d.w. plant material) and in traces in *V. orchidea*. Regarding the flavonoid aglycones, apigenin was identified in both *V. officinalis* and *V. orchidea* unhydrolyzed samples and just in the hydrolyzed extract of *V. teucrium*. The richest species in luteolin was *V. officinalis* (36.48 ± 0.18 μg/g d.w. plant material), the aglycone being present in both hydrolyzed (36.48 ± 0.18 μg/g d.w. plant material) and unhydrolyzed (89.72 ± 3.08 μg/g d.w. plant material) samples, whilst in *V. teucrium* and *V. orchidea* it was only present in glycosylated forms. Different flavone aglycones luteolin, apigenin, chrysoeriol, scutellarein and isoscutellarein have been previously detected in *Veronica* species [29]. Our results are also in line with previous findings by Beara *et al*. [12], who reported the presence of several glycosylated forms of apigenin and luteolin in aerial parts of *Veronica* species, but regarding *V. teucrium* the presence of apigenin was not detected in the unhydrolyzed extract.

### 2.2. HPLC-MS Analysis of Methoxylated Flavones

The importance of methoxylated flavones has increased nowadays due to their superior cancer chemopreventive role. Some of the methoxylated citrus flavonoids have also, in preliminary studies, demonstrated antiproliferative properties [29]. Other compounds like hispidulin have been proven as potent antiepileptic [30] and antifungal agents [31]. Within this frame, another aim of this study was to quantitatively characterize the aerial parts of *V. officialis*, *V. teucrium* and *V. orchidea* related to their jaceosidin, hispidulin, eupalitin, eupatorin, casticin and acacetin content by using a newly developed LC-MS/MS method. Because of the lack of authentic standards, flavonol glycosides are in most cases hydrolysed to their respective aglycons prior to analysis. In this case the samples were subjected to analysis before and after acid hydrolysis.

**Figure 2:**
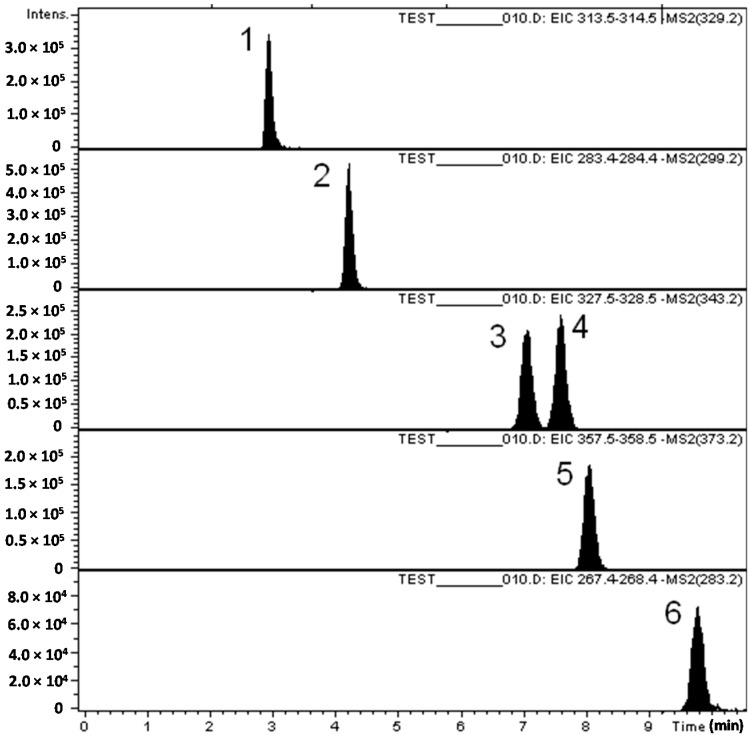
Tipical chromatogram of the six analyzed flavones: (1) jaceosidin; (2) hispidulin; (3) eupalitin; (4) eupatorin; (5) casticin and (6) acacetin.

Under the described chromatographic conditions, the compounds eluted in less than 10 min: jaceosidin (*R*_T_ = 2.9 min), hispidulin (*R*_T_ = 4.2 min), eupalitin (*R*_T_ = 7.05 min), eupatorin (*R*_T_ = 7.6 min), casticin (*R*_T_ = 8.05 min) and acacetin (*R*_T_ = 9.8 min), as seen in Figure 2. The ions monitorized in the MS method are presented in Table 2. Because in the ionization conditions, all flavones have lost a proton, the parent ions detected are always in the form [M − H]^−^. In the process of mass-spectrometry, the pseudo-molecular ions of the flavones (329.3 for jaceosidin, 299.2 for hispidulin, 343.3 for eupatilin, 343.3 for eupatorin, 373.3 for casticin and 283.3 for acacetin) have been fragmented, and based on their daughter ions from the MS spectrum (Table 2) the extracted chromatograms of each compound were constructed for quantification, taking into account the intensity of major ions in the mass spectrum.

**Table 2:** Characteristic ions of standard sterols in full scan and specific ions used in quantification.

Compound	*R*_T_ (min)	*M*	Specific ions for identification Ion [M − H]^−^ > Ion from spectrum
Jaceosidin	2.9	330.3	329.3 > 314
Hispidulin	4.2	300.2	299.2 > 284
Eupatilin	7.05	344.3	343.3 > 328
Eupatorin	7.6	344.3	343.3 > 328
Casticin	8.05	374.3	373.3 > 358
Acacetin	9.8	284.3	283.3 > 268

Hispidulin was found in all samples of *V. officinalis* and *V. orchidea* and only in the hydrolyzed extract of *V. teucrium*. The richest source of hispidulin was *V. orchidea* (4.115 ± 0.09 μg/g d.w. plant material) followed by *V. officinalis* (0.747 ± 0.01 μg/g d.w. plant material). The increased yields of these compounds after acid hydrolysis suggest that they are present also in glycosylated forms. Eupatorin was quantified in both samples of *V. officinalis,* while it was present just in the hydrolyzed sample of *V. teucrium* (0.68 ± 0.04 μg/g d.w. plant material) as seen in Table 3. As a peculiarity, eupatilin was found only in *V. orchidea* extracts (0.491 ± 0.04 μg/g d.w. plant material and 0.252 ± 0.02 μg/g d.w. plant material, respectively). More than that, hispidulin, eupatilin and eupatorin were detected for the first time in *Veronica* genus and the analyzed plant species, respectively.

**Table 3:** The methoxylated flavones content in the analyzed samples (μg/g d.w. plant material).

Polyphenolic Compound	*V. officinalis*	*V. officinalis* ^H^	*V. teucrium*	*V. teucrium* ^H^	*V. orchidea*	*V. orchidea* ^H^
Jaceosidin	–	–	–	–	–	–
Hispidulin	0.747 ± 0.01	2.41 ± 0.08	–	1.476 ± 0.05	4.115 ± 0.09	19.97 ± 1.24
Eupatilin	–	–	–	–	0.491 ± 0.04	0.252 ± 0.02
Eupatorin	0.295 ± 0.02	0.521 ± 0.01	–	0.68 ± 0.04	–	–
Casticin	–	–	–	–	–	–
Acacetin	–	–	–	–	–	–

Note: ^H^—hydrolyzed sample. Values are the mean ± SD (*n* = 3).

### 2.3. HPLC-MS Analysis of Phytosterolic Compounds

In the proposed chromatographic conditions, the compounds eluted with the following retention times: 3.2 min for ergosterol, 3.9 min for brassicasterol, 4.9 min for stigmasterol and campesterol (co-elution) and 5.7 min for β-sitosterol. The ions monitorized in the MS method are presented in Table 4. Because, in the ionization conditions, all sterols have lost a water molecule, the ions detected are always in the form [M − H_2_O + H]^+^ [32].

**Table 4:** Characteristic ions of standard sterols in full scan and specific ions used in quantification.

Compound	*R*_T_	*M*	[M − H_2_O + H]^+^	Specific ions for identification Ion [M − H_2_O + H]^+^ > Ions from spectrum
Ergosterol	3.2	396	379	379 > 158.9; 184.9; 199; 213; 225; 239; 253; 295; 309; 323
Brassicasterol	3.9	398	381	381 > 201.3; 203.3; 215.2; 217.3; 241.2; 255.3; 257.4; 271.1; 297.3; 299.3
Stigmasterol	4.9	412	395	395 > 255; 297; 283; 311; 241; 201
Campesterol	4.9	400	383	383 > 147; 149; 161; 175; 189; 203; 215; 229; 243; 257
β-Sitosterol	5.7	414	397	397 > 160.9; 174.9; 188.9; 202.9; 214.9; 243; 257; 287.1; 315.2

The derived ions of the sterols (379 for ergosterol, 381 for brassicasterol, 395 for stigmasterol, 383 for campesterol and 397 for β-sitosterol) have been fragmented, and based on their daughter ions from the MS spectrum the extracted chromatograms of each compound were constructed. The method can also be applied for quantitative analysis because the intensity of ions in the mass spectrum is proportional to the concentration of the substance in the sample.

For quantitative analysis of the five sterols from *Veronica* species, the extracted chromatograms for each compound were constructed, taking into account the intensity of major ions in the mass spectrum (Table 4) as seen in Table 5.

**Table 5:** The content in sterols (μg/g d.w. plant material) of *Veronica* species extracts.

Phytosterol	*V. officinalis*	*V. teucrium*	*V. orchidea*
β-Sitosterol	729.43 ± 9.21	587.14 ± 5.34	694.47 ± 7.45
Campesterol	30.32 ± 0.31	25.31 ± 0.21	32.75 ± 1.05
Stigmasterol	42.09 ± 1.01	29.16 ± 1.06	25.38 ± 1.28
Ergosterol	1.91 ± 0.01	–	–
Brassicasterol	–	–	–

Note: NF—not found, below limit of detection. Values are the mean ± SD (*n* = 3).

β-Sitosterol, campesterol and stigmasterol were quantified in all the analyzed species. The major sterolic compound was β-sitosterol in all samples, the richest source being *V. officinalis* (729.43 ± 9.21 μg/g d.w. plant material). *V. orchidea* and *V. officinalis* presented similar amounts of campesterol (32.75 ± 1.05 and 30.32 ± 0.31 μg/g d.w. plant material, respectively). The highest amount of stigmasterol was found in *V. officinalis* (42.09 ± 1.01 μg/g d.w. plant material) followed by *V. teucrium* (29.16 ± 1.06 μg/g d.w. plant material). As a peculiarity, ergosterol was found only in *V. officinalis* and in a small amount (1.91 ± 0.01 μg/g d.w. plant material). This is the first report that deals with the quantification of several phytosterols from the *Veronica* genus, respectively from *V. officinalis*, *V. teucrium* and *V. orchidea* aerial parts.

### 2.4. Determination of Phenolic Compound Content

**Figure 3:**
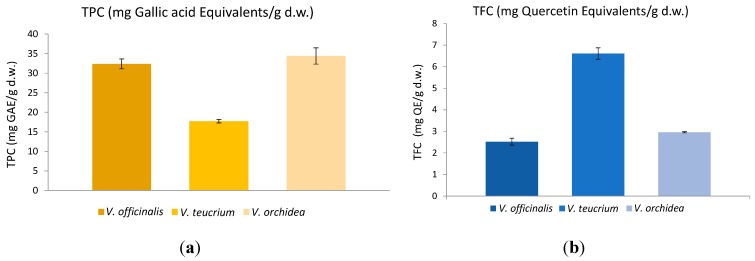
(**a**) Total phenol content (TPC) and (**b**) total flavonoid content (TFC) of *Veronica* species (the bars indicate mean ± SD).

Besides the identified compounds, many other polyphenolic compounds are widely distributed in aerial parts of *Veronica* and contribute to their antioxidant activity. In this part a preliminary comparative view of the total phenolic and flavonoid content of *V. officinalis*, *V. teucrium* and *V. orchidea* is presented. The total phenolic content (TPC; Figure 3a) varied from 17.71 ± 0.44 for *V. teucrium* to 34.40 ± 2.08 mg Gallic acid (GAE) equivalents/g d.w. vegetal material for *V. orchidea*, *V. officinalis* presenting a similar value in terms of TPC to that of *V. orchidea* (32.37 ± 1.27 mg GAE/g d.w.).

The highest total flavonoid content (TFC; Figure 3b) was registered for *V. teucrium* (6.60 ± 0.26 mg Quercetin (QE) equivalents/g d.w. vegetal material). Lower amounts were obtained for *V. orchidea* (2.95 ± 0.02 mg QE/g d.w.) and *V. offcinalis* (2.52 ± 0.16 mg QE/g d.w.). Available literature on *Veronica* species illustrates a high variation of total phenolic contents. While some reported that aerial parts of Serbian *V. teucrium* provides phenolic contents of approx. 157 mg GAE/g of dry methanol extract and 172 mg GAE/g of dry 70% aqueous acetone extract, respectively [33], others reported higher levels in *V. officinalis* with values of approx. 200.20 mg GAE/g of dry methanolic extract [34]. In the present study, the phenolic contents of *Veronica* species were expressed as mg/g of d.w. herbal products. Taking all this into account, the present results are hard to compare with previous results obtained by other authors due to the fact that the authors expressed their values in a different manner.

### 2.5. Antioxidant Features

**Figure 4:**
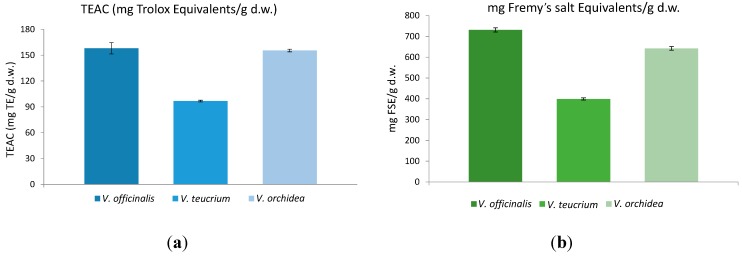
(**a**) Trolox equivelents antioxidant capacity (TEAC) and (**b**) antioxidant activity measured with EPR spectroscopy using Fremy’s salt as a stable synthetic radical of *Veronica* species (the bars indicate mean ± SD).

**Figure 5:**
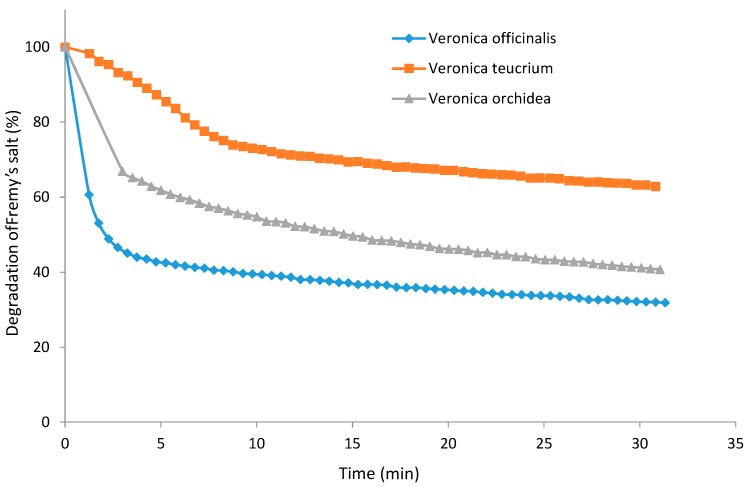
Degradation kinetics of the free radical Fremy’s salt by *V. officinalis*, *V. teucrium* and *V. orchidea*.

Antioxidant activity of natural products, resulting from many compounds such as phenolic compounds (e.g., flavonols) and pigments, offers preventive potential against oxidative stress in the human body. Thus, the trend is towards herbal supplements or foods with more phytochemicals and natural antioxidants. In this part of the investigation, the goal was to evaluate the antioxidant activity of *V. officinalis*, *V. teucrium* and *V. orchidea*. As several authors suggest, the antioxidant capacity cannot be evaluated by using a single test [35,36]. Thus, the widely used TEAC assay was applied for characterizing the free radical scavenging effects of the three *Veronica* species (Figure 4a). Additionally, the antioxidants in speedwell 70% ethanolic extracts were assessed using electron spin resonance (ESR) spectrometry to evaluate their efficiency to reduce a synthetic free radical species, *i.e.*, the semi-stable nitroxide radical Fremy’s salt (potassium nitrosodisulfonate) (Figure 4b and Figure 5).

The TEAC assay was carried out for all *Veronica* species. *V. officinalis* (157.99 ± 6.58 mg TE/g d.w.) and *V. orchidea* (155.41 ± 1.58 mg TE/g d.w.) exhibited similar antioxidant capacities (*p* > 0.05) and higher values (*p* < 0.001) in comparison to *V. teucrium* (96.67 ± 0.26 mg TE/g d.w.). Data about antioxidant activity of *Veronica* species is scarce. However, in a report by Harput *et al*. [34], a comparison study between fourteen *Veronica* species indicated *V. officinalis* had the highest antioxidant activity.

To support the results from the TEAC and Folin-Ciocalteu assays, the three *Veronica* species were additionally analyzed using EPR with Fremy’s salt as stable radical. An important advantage of this assay is the matrix-independent measurement of the reaction between potential antioxidants and radicals in an electromagnetic field instead of the absorbance of light.

The degraded amount of Fremy’s salt after 30 min of incubation time ranged between 730.88 ± 1.82 mg FSE/g d.w. for *V. officinalis* and 398.90 ± 0.99 mg FSE/g d.w. indicating a 68.17% of degradation for the first and just a 37.21% for the latter. Information about the antioxidant activity of *Veronica* species using the electron paramagnetic resonance (EPR) spectrometry assay is not available, so far. Therefore, a comparison with data from other researchers is lacking.

The antioxidant activity of all analyzed *Veronica* species evaluated by TEAC assay and EPR assay was also in line with their corresponding total phenolic content, indicating *V. officinalis* and *V. orchidea* were superior sources of antioxidant compounds (*p* < 0.001).

### 2.6. Antimicrobial Activity

The results of antibacterial activity from *V. officinalis*, *V. teucrium* and *V. orchidea* ethanolic extracts and standard antibiotic streptomycin, tested by microdilution assay, are presented in Table 6 and Table 7.

In the case of *V. officinalis*, the most sensitive bacterial strains were *Listeria monocytogenes* and *Listeria ivanovii*, with equal values of MIC and MBC of 7.81 mg/mL. Regarding *V. teucrium* antibacterial activity, the strains of *Staphylococcus aureus*, *Bacillus cereus*, *Enterococcus faecalis* showed the same values for MIC and MBC (7.81 mg/mL), being the most sensitive. Referring to the *V. orchidea* extract, the most sensitive strains were *Listeria monocytogenes* and *Listeria ivanovii* with equal MICs of 3.9 mg/mL and MBCs of 7.81 mg/mL.

**Table 6:** Minimum inhibitory concentration (MIC) of *V. officinalis*, *V. teucrium* and *V. orchidea* extracts and streptomycin against bacterial strains tested with the microdilution method.

Bacterial Strains	Standard Antibiotic	Minimum Inhibitory Concentration (mg/mL)		
	Streptomycin	*V. officinalis*	*V. teucrium*	*V. orchidea*
*Staphylococcus aureus*	0.03	7.81	7.81	15.62
*Bacillus cereus*	0.015	15.62	7.81	7.81
*Listeria monocytogenes*	0.015	7.81	7.81	3.9
*Listeria ivanovii*	0.06	7.81	7.81	3.9
*Pseudomonas aeruginosa*	0.06	15.62	7.81	15.62
*Enterococcus faecalis*	0.12	7.81	7.81	7.81
*Salmonella typhimurium*	0.06	7.81	7.81	15.62
*Escherichia coli*	0.12	15.62	7.81	7.81

**Table 7:** Minimum bactericidal concentration (MBC) of *V. officinalis*, *V. teucrium* and *V. orchidea* extracts and streptomycin against bacterial strains tested by microdilution method.

Bacterial Strains	Standard Antibiotic	Minimum Bactericidal Concentration (mg/mL)		
	Streptomycin	*V. officinalis*	*V. teucrium*	*V. orchidea*
*Staphylococcus aureus*	0.06	15.62	7.81	31.25
*Bacillus cereus*	0.03	15.62	7.81	7.81
*Listeria monocytogenes*	0.03	7.81	15.62	7.81
*Listeria ivanovii*	0.12	7.81	15.62	7.81
*Pseudomonas aeruginosa*	0.12	31.25	15.62	31.25
*Enterococcus faecalis*	0.24	15.62	7.81	15.62
*Salmonella typhimurium*	0.12	15.62	15.62	31.25
*Escherichia coli*	0.24	31.25	15.62	31.25

The antibacterial activity of sterolic compounds like β-Sitosterol and campesterol has already been demonstrated by Sharma and Mahmood *et al*. [37,38], and these compounds exhibited a strong antibacterial activity on gram-positive bacteria *i.e.*, *S. aureus* and *S. viridians* and a moderate response against gram-negative bacteria like *Escherichia coli*. As a result of these findings, the activity of *Veronica* ethanolic extracts against gram-positive bacteria like *L. monocytogenes*, *L. ivanovii* and *S. aureus* could be attributed at least in part to their high β-Sitosterol content but also to the presence of campesterol and stigmasterol. Nevertheless, other constituents of the extract should also be taken into consideration for the global activity, e.g., the high amounts of apigenin, luteolin and their glycosides [28]. The antibacterial activity of *Veronica* extracts might be influenced also by the presence of hispidulin, as already suggested by Exarchou *et al.* [31]. The most resistant species to the *V. officinalis* extract were *Bacillus cereus*, *Pseudomonas* *aeruginosa* and *Escherichia coli* with equal values of MIC = 15.62 mg/mL and a MBC = 15.62 mg/mL for *B. cereus* and MBC = 31.25 mg/mL for *P. aeruginosa* and *E. coli*. The same bacterial strains were more sensitive to the *V. teucrium* extract, presenting lower values for their MIC and MBC than *V. officinalis* extract. As shown in Table 6 and Table 7, streptomycin has greater activity than the tested extract.

The obtained results for antibacterial activity are comparable to those previously published for *V. montana* water extract [39], and also with the ones obtained for *V. urticifolia* methanolic extract [28], the most sensitive strains in all these cases being *S. aureus* and *L. monocytogenes* whilst the most resistant were by *B. cereus* and *P. aeruginosa*.

## 3. Experimental Section

### 3.1. Plant Collection and Sample Preparation

The plant material (Voucher No. 3592, 3593 and 3594) was collected from Romanian spontaneous flora, in the summer of 2014. Voucher specimens were deposited in the Department of Pharmaceutical Botany Herbarium of the Faculty of Pharmacy, “Iuliu Hațieganu” University of Medicine and Pharmacy, Cluj-Napoca, Romania. The vegetal material was dried in a shaded place and stored properly, until the moment of analysis. Two grams were weighed, ground and extracted with 20 mL of 70% ethanol, twice for 30 min in an ultrasonic bath at room temperature. The samples were then centrifuged at 4000 rpm for 30 min, and the supernatant was recovered, filtered through a 0.45 μm micropore membrane (PTFE, Waters, Milford, MA, USA) and subjected to further analysis.

In order to obtain more accurate data on flavonoid glycosides and aglycones concentration, each sample was analyzed before and after acid hydrolysis. Two milliliters of extractive solution were treated with 2 M hydrochloric acid (2 mL) and ascorbic acid solution (0.2 mL, 100 mg∙mL^−1^), and the mixtures were heated at 80 °C on a water bath for 30 min, ultrasonicated for 15 min, and heated for another 30 min at 80 °C. During the heating, 70% ethanol (1 mL) was added to the extraction mixture every 10 min, in order to ensure the permanent presence of solvent. The mixtures were centrifuged at 4000 rpm and the solutions were diluted with distilled water in a 10 mL volumetric flask and filtered through a 0.45 μm filter before injection [26,32].

For the antimicrobial activity evaluation, the obtained extract was evaporated to dryness under reduced pressure at <30 °C and re-suspended in 1 mL of bi-distilled water.

### 3.2. Chemicals

Chlorogenic acid, *p*-coumaric acid, caffeic acid, rutin, apigenin, quercetin, isoquercitrin, quercitrin, hyperoside, kaempferol, myricetol, fisetin from Sigma (St. Louis, MO, USA), ferulic acid, sinapic acid, gentisic acid, gallic acid, patuletin, luteolin from Roth (Karlsruhe, Germany), cichoric acid, caftaric acid from Dalton (Toronto, ON, Canada), β-sitosterol, brassicasterol, stigmasterol, campesterol and ergosterol from Sigma (St. Louis, MO, USA). HPLC grade methanol, acetonitrile, ethanol, analytical grade orthophosphoric acid, hydrochloric acid and Folin-Ciocâlteu reagent were purchased from Merck (Darmstadt, Germany), ABTS (2,2′-azinobis-3-ethylbenzotiazoline-6-sulphonic acid), Fremys’s salt, sodium carbonate, anhydrous aluminum chloride and resazurin were from Sigma-Aldrich (Steinheim, Germany). Trolox (6-hydroxy-2,5,7,8-tetramethylchroman-2-carboxylic acid) was obtained from Alfa-Aesar (Karlsruhe, Germany). All spectrophotometric data was acquired using a Jasco V-530 UV-vis spectrophotometer (Jasco International Co., Ltd., Tokyo, Japan).

### 3.3. HPLC-MS Analysis of Phenolic Compounds

The identification and quantification of polyphenolic compounds was carried out using an Agilent Technologies 1100 HPLC Series system (Agilent, Santa Clara, CA, USA) equipped with G1322A degasser, G13311A binary gradient pump, column thermostat, G1313A autosampler and G1316A UV detector. The HPLC system was coupled with an Agilent 1100 mass spectrometer (LC/MSD Ion Trap SL). For the separation, a reverse-phase analytical column was employed (Zorbax SB-C18 100 × 3.0 mm i.d., 3.5 μm particle) and the work temperature was set at 48 °C. The detection of the compounds was performed in both UV and MS mode. The UV detector was set at 330 nm until 17.5 min, then at 370 nm. The MS system operated using an electrospray ion source in the negative mode. ChemStation and DataAnalysis software from Agilent were used for processing the chromatographic data. The mobile phase was a binary gradient: methanol and acetic acid 0.1% (*v*/*v*). The elution started with a linear gradient, beginning with 5% methanol and ending at 42% methanol, for 35 min; then 42% methanol for the next 3 min. The flow rate was 1 mL∙min^−1^ and the injection volume was 5 μL.

The MS signal was used only for qualitative analysis based on the specific mass spectra of each polyphenol. Later, the MS traces/spectra of the analyzed samples were compared to spectra from library, which allows positive identification of compounds, based on spectral match. The UV trace was used for quantification of identified compounds from MS detection. For all compounds, the limit of quantification was 0.5 μg/mL, and the limit of detection was 0.1 μg/mL. Calibration curves in the 0.5–50 μg/mL range with good linearity (*R*^2^ > 0.999) for a five point plot were used to determine the concentration of polyphenols in the analyzed samples. In all analyzed samples the compounds were identified by comparison of their retention times and recorded electro-spray mass spectra with those of standards in the same chromatographic conditions [23,24,25,26,32].

### 3.4. HPLC-MS Analysis of Methoxylated Flavones

The separation of the methoxylated flavones was achieved using a Zorbax SB-C18 reversed-phase analytical column (100 × 3.0 mm i.d., 3.5 μm particle) operated at 48 °C. The mobile phase consisted in 0.1% (*v*/*v*) acetic acid and methanol with the following gradient: beginning with 45% methanol and ending at 50% methanol, in 8 min, with a flow rate of 0.9 mL/min and an injection volume of 5 μL. Mass spectrometry analysis was performed on an Agilent Ion Trap 1100 VL mass spectrometer with an electrospray ionization (ESI) source, in the negative mode. For the MS analysis the following optimized conditions were used: gas (nitrogen) temperature 325 °C at a flow rate of 12 L/min, nebulizer pressure 60 psi and capillary voltage +2500 V. The ions monitorized in the MS method are presented in Table 2. Because in the ionization conditions all flavones have lost a proton, the parent ions detected are always in the form [M − H]^−^.

The full identification of compounds was performed by comparing the retention times and mass spectra with those of standards in the same chromatographic conditions. To avoid or limit the background interference, the multiple reactions monitoring analysis mode was used instead of single ion monitoring (e.g., MS/MS instead of MS). ChemStation (vA09.03) and DataAnalysis (v5.3) from Agilent, USA were used for the acquisition and analysis of chromatographic data.

### 3.5. HPLC-MS Analysis of the Phytosterols

Compounds were separated using a Zorbax SB-C18 reversed-phase analytical column (100 × 3.0 mm i.d., 5 μm particle) fitted with a guard column Zorbax SB-C18, both operated at 40 °C. Sterols were separated under isocratic conditions using a mobile phase consisting of 10:90 (*v*/*v*) methanol and acetonitrile. The flow rate was 1 mL/min and the injection volume was 5 μL. Mass spectrometry analysis was performed on an Agilent Ion Trap 1100 VL mass spectrometer with atmospheric pressure chemical ionization (APCI) interface. The instrument was operated in positive ion mode. Operating conditions were optimized in order to achieve maximum sensitivity values: gas temperature (nitrogen) 325 °C at a flow rate of 7 L/min, nebulizer pressure 60 psi and capillary voltage −4000 V. The full identification of compounds was performed by comparing the retention times and mass spectra with those of standards in the same chromatographic conditions. To avoid or limit the background interference, the multiple reactions monitoring analysis mode was used instead of single ion monitoring (e.g., MS/MS instead of MS). Calibration curves were obtain from standard solutions at different concentration levels, selected as representative of the range of concentration in the sample. Regression analysis of various concentrations of standard solutions (0.08–8 μg/mL) gave good correlation coefficients for the calibration curves of sterols. The software ChemStation (vA09.03) and DataAnalysis (v5.3) from Agilent, USA were used for the acquisition and analysis of chromatographic data [32].

### 3.6. Determination of Phenolic Compound Content

#### 3.6.1. Determination of Total Phenolic Content

The total phenolic content (TPC) of the extracts was measured using the Folin-Ciocâlteu method with some modifications. Two milliliters from each ethanolic extract were diluted 25 times and then mixed with Folin-Ciocâlteu reagent (1 mL) and distilled water (10 mL) and diluted to 25 mL with a 290 g/Lsolution of sodium carbonate. The samples were incubated in the dark for 30 min. The absorbance was measured at 760 nm, using a Jasco UV-VIS spectrophotometer. A standard curve was prepared by using different concentrations of gallic acid and the absorbances were measured at 760 nm. TPC values were determined using an equation obtained from the calibration curve of gallic acid (*R*^2^ = 0.999). Total polyphenolic content was expressed as mg gallic acid equivalents/g dry material plant (mg GAE/g plant material) [26,40].

#### 3.6.2. Determination of Total Flavonoid Content

The total flavonoid content was calculated and expressed as quercetin equivalents after the method described in the Romanian Pharmacopoeia (10th Edition) for *Cynarae folium*. Each extract (5 mL) was mixed with sodium acetate (5 mL, 100 g/L), aluminum chloride (3 mL, 25 g/L), and made up to 25 mL in a calibrated flask with methanol. Each solution was compared with the same mixture without reagent. The absorbance was measured at 430 nm. The total flavonoid content values were determined using an equation obtained from calibration curve of the quercetin graph (*R*^2^ = 0.999) (Tămaș *et al.*, 2009) [41].

### 3.7. Antioxidant Features

#### 3.7.1. Trolox Equivalent Antioxidant Capacity (TEAC)

The effects of extract on the synthetic ABTS radical were estimated by the method previously described by Toma *et al.* [26]2015 with some modifications. In the Trolox equivalent antioxidant capacity (TEAC) assay, the antioxidant capacity is reflected in the ability of the natural extracts to decrease the color, reacting directly with the ABTS cation radical. The latter was obtained by oxidation of 2,2′-azinobis(3-ethylbenzothiazoline-6-sulfonic acid (ABTS) with potassium persulfate. Original extracts were diluted appropriately, and 3 μL from the diluted extract were added to 997 μL ABTS solution. The amount of ABTS radical consumed by the tested compound was measured at 734 nm, after 30 min of reaction time, with 70% ethanol (vehicle) as negative control. The evaluation of the antioxidant capacity was obtained using the total change in absorbance at this wavelength.

#### 3.7.2. EPR Measurements

EPR measurements were carried out by the method previously described by Mocan *et al.* [24] with some modifications. Appropriate extract dilutions (1:400) were prepared and 25 μL aliquots were allowed to react for 30 min with an equal volume of a solution of Fremy’s salt (1 mM in phosphate buffer, pH 7.4). EPR spectra of Fremy’s radical were obtained with a Bruker Elexsys E500 spectrometer (Bruker, Billerica, MA, USA). The antioxidant activity expressed as mg Fremy’s salt equivalents reduced by 25 μL diluted extract was calculated by comparison with a control reaction with 25 μL Fremy’s salt and 25 μL of extraction solvent.

### 3.8. Antibacterial Activity

#### 3.8.1. Microorganisms and Culture Conditions

For the bioassay eight bacterial strains were used, four Gram positive: *Staphylococcus aureus* (ATCC 49444), *Bacillus cereus* (ATCC 11778), *Listeria monocytogenes* (ATCC 19114), *Listeria ivanovii* (ATCC 19119) and four gram negative: *Pseudomonas aeruginosa* (ATCC 27853), *Enterococcus faecalis* (ATCC 29212), *Salmonella typhimurium* (ATCC 14028) and *Escherichia coli* (ATCC 25922). All of the tested microorganisms were obtained from the Food Biotechnology Laboratory, Life Sciences Institute, University of Agricultural Sciences and Veterinary Medicine Cluj-Napoca, Romania. The bacteria were cultured on Muller-Hinton gar and cultures were stored at 4 °C and subcultured once a month.

#### 3.8.2. Microdilution Method

The modified microdilution technique was used to evaluate antimicrobial activity. Bacterial species were cultured overnight at 37 °C in Tryptic Soy Broth (TSB) medium. The bacterial cell suspensions were adjusted with sterile saline to a concentration of approximately 3 × 10^5^ CFU/mL in a final volume of 100 μL per well. The inoculum was stored at +4 °C for further use. Dilutions of the inoculum were cultured on solid Muller-Hinton (MH) for bacteria to verify the absence of contamination and to check the validity of the inoculum. Determinations of minimum inhibitory concentrations (MICs) were made using a serial dilution technique using 96-well microtitre plates. Different dilutions of ethanolic extract were carried out over the wells containing 100 μL of Tryptic Soy Broth (TSB) and afterwards, 10 μL of inoculum was added to all the wells. The microplates were incubated for 24 h at 37 °C. The MIC of the samples was detected following the addition of 20 μL (0.2 mg/mL) of resazurin solution to each well, and the plates were incubated 2 h at 37 °C. A change from blue to pink indicates reduction of resazurin and therefore bacterial growth. The MIC was defined as the lowest extract concentration that prevented this color change. The minimum bactericidal concentrations (MBCs) were determined by serial subcultivation of a 2 μL into microtitre plates containing 100 μL of broth per well and further incubation for 48 h at 37 °C. The lowest concentration with no visible growth was defined as MBC, indicating a 99.5% kill rate for the original inoculum. Streptomycin (Sigma P 7794, Santa Clara, CA, USA) (0.05–3 mg/mL) was used as positive control for bacterial growth. A 10% solution of ethanol in water was used as negative control.

### 3.9. Statistical Analysis

A statistical approach was designed and the experimental data were evaluated using one-way analysis of variance (ANOVA), with *p* < 0.05 as threshold for statistical significance. The statistical results confirm the hypothesis that the differences between the results are either not significant (*p* > 0.05), significant (0.001 < *p* < 0.05) or highly significant (*p* < 0.001). The average of multiple measurements (triplicates) was listed in the tables together with the standard deviations. Statistical analysis was performed using the Excel software package.

## 4. Conclusions

In the present study, *V. officinalis*, *V. teucrium* and *V. orchidea* were investigated for their chemical composition, antioxidant and antimicrobial effects. These species showed marked phenolic and sterolic content, and among the determined compounds, *p*-coumaric acid, ferulic acid, luteoline, hispidulin and β-sitosterol were dominant. More than that, hispidulin, eupatorin and eupatilin were detected for the first time in the *Veronica* genus. Nevertheless, representatives of *Veronica* genus were never investigated related to their content in phytosterols. *V. officinalis* and *V. orchidea* extracts presented similar antioxidant capacities related to their phenolic content whilst the values registered for *V. teucrium* extract were lower. Concerning the antimicrobial potential of the investigated species, *Staphylococcus aureus*, *Listeria monocytogenes* and *Listeria ivanovii* were the most sensitive strains with MIC values between 3.9 and 15.62 mg/mL. The results of this study bring new perspectives for further pharmaceutical and nutraceutical development of *Veronica* species as natural sources of bioactive compounds with antioxidant and antimicrobial potential. However, further studies are necessary in order to elucidate the mechanisms of *in vivo* antioxidant action, bioavailability and the involved metabolic pathways.
